# Idiopathic, Serial Coronary Vessels Dissection in a Young Woman with Psychological Stress: A Case Report and Review of the Literature

**DOI:** 10.1155/2012/498465

**Published:** 2012-10-23

**Authors:** Alessio Arrivi, Caterina Milici, Carlo Bock, Attilio Placanica, Enrico Boschetti, Marcello Dominici

**Affiliations:** Interventional Cardiology Unit, Santa Maria University Hospital, Via Tristano Di Joannuccio 1, 05100 Terni, Italy

## Abstract

Spontaneous coronary artery dissection (SCAD) is a very rare disease, associated with high mortality rate, whose etiology and pathogenesis are poorly understood. Its sporadic nature and the varied angiographic extent make firm recommendations regarding revascularization impossible. The case described is that of a young, otherwise healthy woman, without a known underlying condition which may lead to SCAD, but with a history of intense psychological stress. We managed the patient with a conservative approach based on watchful waiting, medical therapy, and plain old balloon angioplasty (POBA) with low inflation atmospheres.

## 1. Introduction

Spontaneous coronary artery dissection (SCAD) is a very rare cause of acute myocardial infarction (AMI) in young patients, whose pathology and treatment have not been fully clarified yet. For these reasons the disorder does not find any perfect collocation in cardiac disease manuscripts. 

Several conditions have been associated with SCAD, such as atherosclerosis, connective tissue disorders, and the peripartum episode. Here we describe a complex case of an idiopathic, serial SCAD, in a 38-year-old woman, without any related risk factors.

## 2. Case Presentation

**Figure 1:**
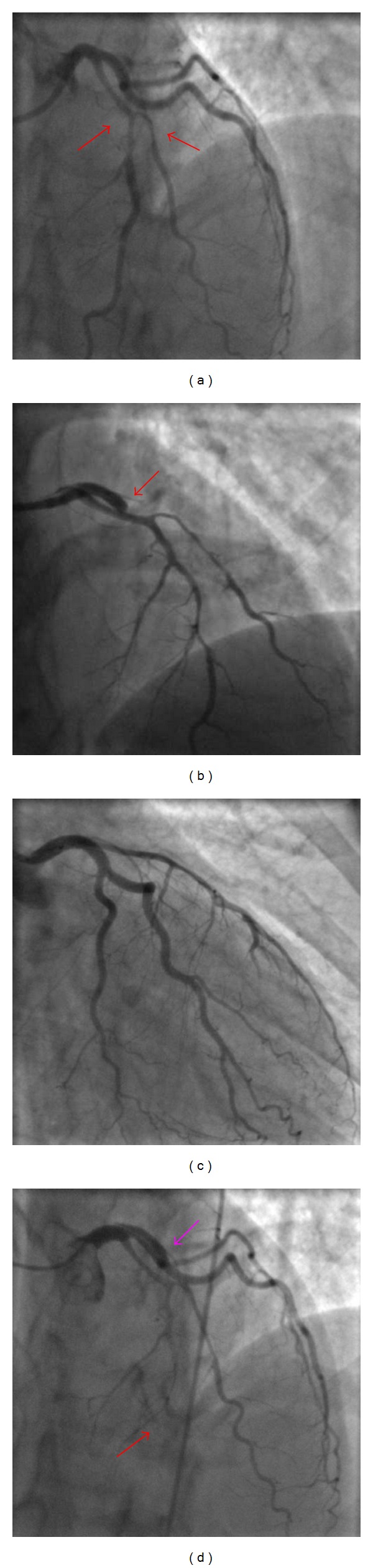
*Angiographic projections* (a) (35°LAO and 23°CRA):  dissection of the left anterior descending (LAD) artery, involving the origin and proximal tract of the first diagonal branch (FDB) (red arrows); (b) (9°RAO and 39°CRA): progression of the dissection to the distal tract of the LAD and in the FDB, and proximal occlusion of the left circumflex artery (LCX) (red arrow); (c) (25°RAO  and 23°CAU): angiographic result after POBA in the LCX; (d) (35°LAO  and 23°CRA): distal occlusion of the LAD (red arrow) and patency of the LCX in spite of the presence of a persistent line of dissection (violet arrow).

A 38-year-old woman, without any cardiovascular risk factors or drug abuse, presented to the emergency department with a 60-minute history of chest pain. She referred no prior medical history but an intense period of psychological stress (she recently applied for a divorce). Electrocardiographic (ECG) findings were suggestive for anterior acute myocardial infarction (AMI). Echocardiography revealed an apical and septal akinesia with an ejection fraction (EF) of 45%. Troponin I, CPK, and CK-MB levels were over the upper normal limit. We administered acetylsalicylic acid 250 mg iv, clopidogrel 300 mg po, heparin bolus 4000 IU iv followed by continuous infusion, ranitidine 50 mg iv, and atenolol 5 mg iv. The coronary angiogram, performed using the left radial approach, revealed a long dissection of the proximal and medium tract of the left anterior descending (LAD) artery, involving the origin and proximal tract of the first diagonal branch (FDB) (TIMI flow I) (Figure 1(a)).

The operator applied the intra-aortic balloon pump (IABP) and administered nitrate ic and GP IIb/IIIa inhibitors. Due to the regression of angina with stable hemodynamic parameters, no further operation was carried out and the woman was transferred, asymptomatic, to the coronary care unit (CCU). 

By the third day, CPK levels normalized, with no more rising during the remainder of the hospitalisation, and the IABP was removed. During the control angiogram, performed seven days later using a 4 French catheter, a progression of the dissection to the distal tract of the LAD and the FDB was shown and a proximal dissection with occlusion of the left circumflex artery (LCX) appeared (Figure 1(b)). Clinical conditions got worse with the renewal of the chest pain. Plain old balloon angioplasty (POBA) was immediately performed, obtaining TIMI flow III and symptoms resolution (Figure 1(c)). No stents were implanted, and the woman was transferred again to the CCU. 

Four days after, the patient complained of a short period of chest discomfort. Despite an unchanged ECG and no enzyme rising, a third angiogram was carried out, showing distal occlusion of the LAD and LCX patency in spite of the presence of a persistent line of dissection (Figure 1(d)). Considering the clinical stable conditions, we decided to manage the patient with a watchful waiting and medical therapy. 

**Figure 2:**
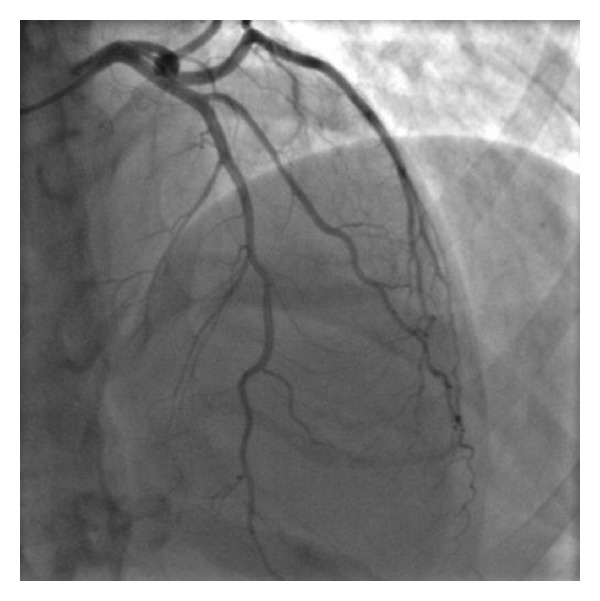
*Final angiographic* (35°LAO and 23°CRA) whereby normal patency of the coronary arteries is seen.

The last control angiogram, performed 36 days after the admission, showed normal patency of all coronary arteries without any line of dissection (Figure 2). So the patient was discharged, and the echocardiogram demonstrated an EF of 50% with residual akinesia of the apex.

At the one-year followup, the patient was in good clinical condition (NYHA I, CCS 0).

## 3. Discussion

Spontaneous coronary artery dissection (SCAD) is a very rare disease with an incidence of 0, 1% among patients who undergo coronary angiography [1]. The real incidence of this condition may be underestimated because of a large number of cases leading to sudden cardiac death before diagnosis [2]. More than 70% of SCAD occur in women, with a predilection for the left coronary artery system, and the mean age at the presentation is 35 to 40 years [3]. Generally, this disease is associated with high mortality, about 50% at presentation [4], with an 85% survival rate for patients who survive the acute phase [5]. The etiology and pathogenesis of SCAD remain poorly understood. Tanis et al. divided SCAD into four groups: peripartum; atherosclerotic; related to other causes (i.e., connective tissue diseases, systemic lupus erythematosus (SLE)), and conditions (such as vigorous exercise, prolonged sneezing, or cocaine abuse); idiopathic [6]. Changes within the arterial wall and/or superimposed abnormal shear forces are implicated. Dissection usually occurs in the outer tunica media or between the media and external elastic lamina. Where intimal tears are not present, intramural hemorrhage from ruptured vasa vasorum is implicated [7]. The clinical presentation depends on the extent and severity of the dissection and ranges from unstable angina to sudden cardiac death. 

The case described is that of a young, otherwise healthy woman, without a known underlying condition which may lead to SCAD, but with a history of intense psychological stress. There is very little published literature about this condition as a trigger for SCAD with only a few cases, without any clear description about the potential role [8, 9]. One possible suggestion as to the pathophysical mechanism may be a result of an excessive sympathetic nervous system activation which may cause shearing stresses on the coronary arteries, disrupting the thin-walled vasa vasorum and causing hemorrhage within the media of the artery. The treatment options for SCAD include medical treatment, percutaneous coronary intervention with stenting, and bypass graft operation [10]. The sporadic nature and the varied angiographic extent of the disease make firm recommendations regarding revascularization impossible. Considering the regression of angina with stable hemodynamic parameters as well as the need for stenting long coronary segments and bifurcations, we initially decided to manage the patient with a watchful waiting and medical therapy. A POBA using low inflation atmospheres was performed with the intent to restore blood flow only when a complete occlusion of the LCX occurred during the second angiogram. Neither the worsening condition of LAD nor the persistent dissection of the LCX at the third angiogram was approached with stenting because of stable haemodynamic parameters. 

The good angiographic result at the last angiogram, performed 36 days after the admission, as well as the stable conditions after 1 year of followup, justified a posteriori the conservative approach used in this context. 

## Abbreviations

LAO: Left anterior oblique
RAO: Right anterior oblique
CRA: Cranial
CAU: Caudal.
